# Baseline characteristics may impact treatment duration of cabazitaxel in patients with mCRPC: a subanalysis of data from a post-marketing surveillance

**DOI:** 10.1093/jjco/hyad128

**Published:** 2023-10-06

**Authors:** Nobuaki Matsubara, Hideyasu Matsuyama, Hirotaka Kazama, Takeshi Seto, Yoshinori Sunaga, Kazuhiro Suzuki

**Affiliations:** Department of Medical Oncology, National Cancer Center Hospital East, Chiba, Japan; JA Yamaguchi Kouseiren Nagato General Hospital, Department of Urology, Yamaguchi, Japan; Medical Affairs, Sanofi K.K., Tokyo, Japan; Medical Affairs, Sanofi K.K., Tokyo, Japan; Medical Affairs, Sanofi K.K., Tokyo, Japan; Department of Urology, Gunma University Graduate School of Medicine, Maebashi, Japan

**Keywords:** cabazitaxel, granulocyte-colony stimulating factor, overall survival, prostate cancer, prostate-specific antigen

## Abstract

**Objective:**

Cabazitaxel has demonstrated improvements in overall survival among patients with metastatic castration-resistant prostate cancer (mCRPC) in the pivotal comparison clinical trials TROPIC, PROSELICA and CARD. However, these trials include mCRPC patients with similar characteristics, and there are limited data on how baseline characteristics affect treatment discontinuation in the patient population.

**Methods:**

To assess individual factors that may impact the discontinuation rate of cabazitaxel treatment, we conducted a *post hoc* analysis of data from a nationwide all-case, post-marketing surveillance of cabazitaxel in Japan. Patients were grouped according to the number of cabazitaxel treatment cycles received (1–2 and ≥3 cycles). Predictive factors were identified through multivariate logistic regression analysis.

**Results:**

Across 660 patients with metastatic castration-resistant prostate cancer, 70.2% received ≥3 cycles of cabazitaxel treatment. Those receiving 1–2 cycles of cabazitaxel had a greater proportion of patients with poorer Eastern Cooperative Oncology Group Performance Status, presence of lung and liver metastases, higher prostate-specific antigen level and prior radiation therapy at baseline. Regardless of the number of cabazitaxel cycles received, the primary reason for discontinuation was progression of disease rather than adverse events. Compared with those receiving 1–2 cycles, a lower proportion of patients receiving 3–10 and ≥11 cycles of cabazitaxel treatment experienced adverse events. Multivariate analysis showed a significant association between early discontinuation and presence of liver lesions, poorer Eastern Cooperative Oncology Group Performance Status and higher prostate-specific antigen level at baseline.

**Conclusions:**

Post-marketing surveillance data suggest physicians should individualize cabazitaxel treatment based on certain patient characteristics at baseline.

## Teaser

This *post hoc* analysis of a post-marketing surveillance in Japan suggests early discontinuation of cabazitaxel treatment may be associated with high disease burden at baseline.

## Background

Metastatic castration-resistant prostate cancer (mCRPC) is a major contributor to mortality among prostate cancer (PC) subtypes, with an average overall survival (OS) of only 16–18 months following disease progression, prompting the urgency to identify a viable treatment option for the patient population (1). Androgen deprivation therapy has been a pivotal treatment option for PC since 2004; patients with metastatic disease show low levels of prostate-specific antigen (PSA) during the early stages of therapy. Unfortunately, many patients eventually face progression within an average of 2–3 years (1).

Docetaxel chemotherapy is the standard first-line treatment for mCRPC (2). However, taxane-based agents have been associated with haematological toxicity and relatively high incidence of febrile neutropenia. Thus, these agents should only be used in mCRPC after careful consideration, to achieve optimal patient outcomes (3, 4). Cabazitaxel has been approved for the treatment of CRPC at a starting dose of 25 mg/m^2^ in Japan and Europe (5), and is administered via infusion once every 3 weeks in combination with oral prednisolone taken daily throughout treatment (6).

Based on several studies, cabazitaxel is effective in improving PSA level, OS and time to treatment failure (TTF) in patients with mCRPC who have received docetaxel previously (7, 8). However, some baseline characteristics of the patients may impact cabazitaxel treatment duration (9). Among patients receiving sequential therapy for mCRPC, a study conducted in Japan observed a negative correlation between OS and PSA levels at baseline with cabazitaxel treatment (10), suggesting cabazitaxel may provide greater survival benefit when initiated in patients with low PSA levels. In a study by Suzuki et al. (11), the cumulative rates of discontinuation and death among patients were 38.6% and 11.7%, respectively, within 3 months, suggesting that death was not the leading cause of early discontinuation. Based on these data, early discontinuation of cabazitaxel may be attributed to inappropriate timing of treatment initiation or intolerability.

Identification of patient factors associated with early discontinuation of cabazitaxel may assist in appropriate treatment decisions (9). To identify potential key features that may increase the likelihood of cabazitaxel discontinuation among patients with mCRPC, we analysed discontinuation rates at different treatment cycles from a post-marketing surveillance (PMS) dataset and took a quantitative approach to detect signs of associations through a multivariate analysis.

## Patients and Methods

This study reports a post hoc analysis of data from a nationwide all-case PMS in which all patients who received cabazitaxel treatment in Japan between September 2014 and June 2015 were registered (11).

During the PMS, case report forms were completed by the investigators before starting cabazitaxel treatment and during each treatment cycle (every 3 weeks) for up to 1 year (11). Collected data included patient demographics, disease characteristics, cabazitaxel exposure and prior and concomitant therapies. Statistical analyses were conducted using the SAS software (version 9.2 or 9.4). To identify factors associated with the number of cabazitaxel cycles received, we performed a multivariate logistic regression analysis with backward selection (significance level, 0.1), which incorporated factors showing significant effects in a univariate analysis. The magnitude of associations was assessed by estimating the odds ratios (ORs) with their 95% confidence intervals (CIs).

Early discontinuation was defined as discontinuation of cabazitaxel during the first or second cycle of treatment, regardless of its reason. This cut-off was determined from previous reports that suggest treatment discontinuation during 1–2 cycles was mainly attributed to tolerability issues (9). Patients were grouped according to the total number of cabazitaxel treatment cycles received (1–2 and ≥3 cycles). To identify the reasons for treatment discontinuation associated with longer duration of cabazitaxel treatment, further analyses were conducted in patients who received ≥11 cycles of cabazitaxel treatment (9).

Adverse events (AEs) were coded using terminology from the Medical Dictionary for Regulatory Activities (MedDRA) version 12.0/16.0 (Japanese translation).

## Results

Across the 660 patients with mCRPC who were registered in a Japanese PMS study of cabazitaxel (11), the number of cabazitaxel cycles received were 1–2 cycles for 197 patients (29.8%) and ≥3 cycles for 463 patients (70.2%). In Japan, the approved dosage of cabazitaxel is 25 mg/m^2^. A lower proportion of patients who received ≥3 cycles of cabazitaxel were given a mean dose of cabazitaxel ≥25 mg/m^2^, compared with patients who received 1–2 cycles (15.3% and 23.9%, respectively) (Table 1a). The proportion of patients receiving the initial dose of ≥25 mg/m^2^ was comparable between patients who underwent 1–2 cycles or ≥3 cycles of cabazitaxel (29.4% versus 30.5%). In contrast, a greater proportion of patients who underwent 1–2 cycles of cabazitaxel received an initial dose of ≥15 and <20 mg/m^2^ compared with those who underwent ≥3 cycles of cabazitaxel (19.3% versus 17.1%, respectively) (Table 1b).

**Table 1a:** Mean dose of cabazitaxel by number of cycles received

Mean dose of cabazitaxel (mg/m^2^/cycle), *n* (%)	Cabazitaxel cycles received	
	1–2 (*n* = 197)	≥3 (*n* = 463)
<15	4 (2.0)	11 (2.4)
≥15 and <20	44 (22.3)	107 (23.1)
≥20 and <25	102 (51.8)	274 (59.2)
≥25	47 (23.9)	71 (15.3)

**Table 1b:** Initial dose of cabazitaxel by number of cycles received

Initial dose of cabazitaxel (mg/m^2^/cycle), *n* (%)	Cabazitaxel cycles received	
	1–2 (*n* = 197)	≥3 (*n* = 463)
<15	4 (2.0)	13 (2.8)
≥15 and <20	38 (19.3)	79 (17.1)
≥20 and <25	97 (49.2)	230 (49.7)
≥25	58 (29.4)	141 (30.5)

Patient baseline demographics were consistent across the two groups (Table 2). The median age of patients receiving 1–2 cycles and ≥3 cycles of cabazitaxel was 71 and 70 years, respectively, with a majority of patients being between the ages of 65 and 75 years (54.3% and 51.6%, respectively). Most patients in both groups had an Eastern Cooperative Oncology Group performance status (ECOG PS) of 0 or 1. At baseline, median PSA levels were 228.4 ng/mL among patients who discontinued cabazitaxel treatment earlier, versus 145.5 ng/mL who discontinued after ≥3 cycles.

**Table 2:** Baseline characteristics by number of cabazitaxel cycles received

Characteristics	Cabazitaxel cycles received	
	1–2 (*n* = 197)	≥3 (*n* = 463)
Age, years, median (range)	71 (55–88)	70 (43–91)
Age breakdown, *n* (%) 65 years ≥65 and <75 years ≥75 years Missing	34 (17.3) 107 (54.3) 55 (27.9) 1 (0.5)	99 (21.4) 239 (51.6) 125 (27.0) 0
Body weight, kg, mean (SD)	60.2 (10.1)	61.9 (10.6)
Disease duration, years, median (range)	3.3 (0.6–12.3)	4.6 (0.5–19.8)
ECOG PS, *n* (%) 0 1 2 3 4 Missing	103 (52.3) 62 (31.5) 22 (11.2) 9 (4.6) 1 (0.5) 0	309 (66.7) 132 (28.5) 13 (2.8) 8 (1.7) 0 1 (0.2)
Gleason score, *n* (%) 2–7 8–10 Missing	29 (14.7) 157 (79.7) 11 (5.6)	75 (16.2) 359 (77.5) 29 (6.3)
TNM classification, *n* (%) T1 + T2 T3 + T4 + TX T missing N0 N1 + NX N missing M0 M1 + MX M missing	29 (14.7) 167 (84.8) 1 (0.5) 86 (43.7) 109 (55.3) 2 (1.0) 60 (30.5) 135 (68.5) 2 (1.0)	84 (18.1) 371 (80.1) 8 (1.7) 211 (45.6) 250 (54.0) 2 (0.4) 130 (28.1) 331(71.5) 2 (0.4)
Metastatic sites, *n* (%) Bone Prostate Regional lymph node Distant lymph node Liver Seminal vesicles Lung Bladder Other None	176 (89.3) 140 (71.1) 85 (43.1) 56 (28.4) 40 (20.3) 27 (13.7) 28 (14.2) 21 (10.7) 16 (8.1) 3 (1.5)	405 (87.5) 326 (70.4) 181 (39.1) 127 (27.4) 48 (10.4) 51 (11.0) 42 (9.1) 44 (9.5) 20 (4.3) 3 (0.6)
Palliative radiation therapy, *n* (%) No Yes Spine Other than spine	134 (68.0) 63 (32.0) 42 (21.3) 38 (19.3)	329 (71.1) 134 (28.9) 68 (14.7) 90 (19.4)
Most recent therapy, *n* (%) Docetaxel Enzalutimide or abiraterone Others	52 (26.4) 139 (70.6) 6 (3.1)	80 (17.3) 372 (80.3) 11 (2.4)
Prophylactic G-CSF, *n* (%)	134 (68.0)	403 (87.0)
PSA, ng/mL, mean (SD)	637.0 (1513.8)	442.9 (1033.6)

ECOG PS, Eastern Cooperative Oncology Group performance status; G-CSF, granulocyte-colony stimulating factor; PSA, prostate-specific antigen; SD, standard deviation; TNM, tumour, nodes, metastases.

Patients receiving 1–2 cycles of cabazitaxel had a greater proportion of patients with poorer ECOG PS compared with those receiving ≥3 cycles of cabazitaxel (ECOG PS of ≥2 in 16.2% and 4.5%, respectively). Patients who discontinued treatment during cycles 1–2 tended to receive radiation therapy for the spine at baseline, compared with those receiving ≥3 cycles (21.3% versus 14.7%). Patients who discontinued early tended to have liver (20.3% versus 10.4% in ≥3 cycles) and lung metastases (14.2% versus 9.1% in ≥3 cycles) at baseline. The proportions of patients who received docetaxel as most recent prior therapy were 26.4% and 17.3% among patients receiving 1–2 and ≥ 3 cycles of cabazitaxel, respectively.

Among patients receiving 1–2 cycles of cabazitaxel, 68.0% received prophylactic granulocyte-colony stimulating factor (G-CSF), while 87.0% who had undergone ≥3 cycles received prophylactic G-CSF.

Results from univariate and multivariate analyses are shown in Table 3. In the univariate analysis, patients with liver lesions at baseline had significantly greater odds of discontinuing cabazitaxel treatment earlier, relative to those without (OR, 2.20; 95% CI, 1.39–3.48). The same was true for patients with poorer ECOG PS whose odds for discontinuing earlier were 1.84 higher than those with better ECOG PS (95% CI, 1.31–2.59). The odds of discontinuing earlier were 1.55 times greater among patients with higher PSA level (≥164.9) versus lower PSA level (<164.9) (95% CI, 1.11–2.18).

**Table 3:** Univariate and multivariate analyses of potential predictors for early discontinuation of cabazitaxel treatment

Characteristics	Patients (*N*)	Cabazitaxel cycles received (1–2), *n* (%)	Unadjusted OR (95% CI)^a^	*P* value	Adjusted OR (95% CI)^b^	*P* value
Age	660	197 (29.85)				
<71 years	331	94 (28.40)	Ref	0.45		
≥71 years	328	102 (31.10)	1.14 (0.81–1.59)			
Unknown	1	1 (100.00)	–			
Gleason score						
Well differentiated (2–4)	6	4 (66. 67)	Ref	0.13	Ref	0.089
Moderately differentiated (5–7)	98	25 (25.51)	0.17 (0.03–0.99)		0.08 (0.01–0.78)	
Poorly differentiated (8–10)	516	157 (30.43)	0.22 (0.04–1.21)		0.10 (0.01–0.92)	
Missing	40	11 (27.50)	–			
Presence of liver lesion						
No	572	157 (27.45)	Ref	<0.001	Ref	<0.001
Yes	88	40 (45.45)	2.20 (1.39–3.48)		2.43 (1.45–4.09)	
ECOG PS						
0	412	103 (25.00)	Ref	<0.001	Ref	0.007
1–4	247	94 (38.06)	1.84 (1.31–2.59)		1.72 (1.16–2.55)	
Missing	1	0 (0.00)	–		–	
PSA level, ng/mL						
<164.9	327	83 (25.38)	Ref	0.010	Ref	0.004
≥164.9	327	113 (34.56)	1.55 (1.11–2.18)		1.78 (1.20–2.64)	
Missing	6	1 (16.67)	–		–	
Neutrophil count, /mm^3^						
<4500.0	296	85 (28.72)	Ref	0.57		
≥4500.0	298	92 (30.87)	1.11 (0.78–1.58)			
Missing	66	20 (30.30)	–			
Pre-treatment with curative local therapy						
Yes	212	64 (30.19)	Ref	0.86		
No	447	132 (29.53)	0.97 (0.68–1.38)			
Missing	1	1 (100.00)	–			
Pre-treatment with palliative radiotherapy						
Yes	197	63 (31.98)	Ref	0.44		
No	463	134 (28.94)	0.87 (0.60–1.24)			
Missing	0	0 (0.00)	–			
Combination treatment with palliative radiotherapy						
Yes	20	3 (15.00)	Ref	0.15		
No	640	194 (30.31)	2.46 (0.71–8.51)			
Missing	0	0 (0.00)	–			
Dosing frequency of docetaxel before treatment (times)						
<10 ≥10 Missing	321 309 30	101 (31.46) 84 (27.18) 12 (40.00)	Ref 0.81 (0.58–1.15) –	0.24		
Reason for discontinuation of chemotherapy PD AE + others Missing	534 108 18	162 (30.34) 27 (25.00) 8 (44.44)	Ref 0.77 (0.48–1.23) –	0.27		
Initial dose of cabazitaxel (kg/m^2^) <20 ≥20	417 243	124 (29.74) 73 (30.04)	Ref 1.01 (0.72–1.43)	0.93		

AE, adverse event; CI, confidence interval; ECOG PS, Eastern Cooperative Oncology Group performance status; OR, odds ratio; PD, progressive disease; PSA, prostate-specific antigen; Ref, reference.

^a^Univariate logistic analysis.

^b^Multivariate logistic analysis with backward selection included Gleason score, presence of liver lesions, Eastern Cooperative Oncology Group performance status and PSA levels.

Factors that maintained significance for early discontinuation of cabazitaxel treatment in the multivariate analysis were the presence of liver lesions, poorer ECOG PS and higher PSA level (Table 3).

**Figure 1:**
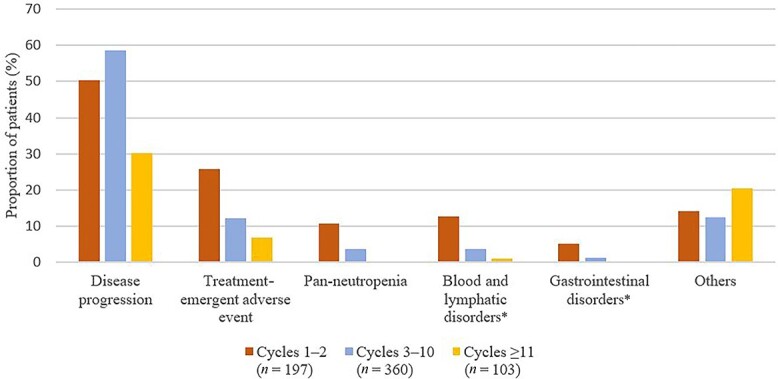
Reasons for treatment discontinuations, stratified by the number of cabazitaxel treatment cycles received. *Defined according to MedDRA system organ class.

The most prevalent reason for discontinuation was progression of disease (Fig. 1).

This was followed by treatment-emergent AEs in patients receiving 1–2 cycles of cabazitaxel, and ‘others’ (patient request and physician decision) in those receiving ≥3 cycles.

In patients receiving 1–2 cycles of cabazitaxel, 10.7% of patients discontinued treatment due to pan-neutropenia (composite of neutropenia, febrile neutropenia and decreased neutrophil count), 12.7% due to blood and lymphatic disorders (MedDRA SOC) and 5.1% due to gastrointestinal disorders (MedDRA SOC). Proportions for these AEs were lower in patients receiving 3–10 (3.6%, 3.6%, and 1.4%) and ≥11 cycles of cabazitaxel (0.0%, 1.0%, and 0.0%), respectively.

## Discussion

Our study showed that early treatment discontinuation (cycles 1–2) versus later (≥3 cycles) had a greater proportion of patients with the following characteristics at baseline: high median PSA levels, poor ECOG PS, radiation therapy for bone metastasis, liver and lung metastasis lesions and taxane treatment as most recent prior therapy.

Some patients did not receive G-CSF as a primary prophylactic because they were recruited during the early stages of cabazitaxel approval in Japan, at which time prophylactic administration of G-CSF was only recommended for vulnerable patients. Among patients who did not receive prophylactic G-CSF, there was a higher proportion of patients discontinuing cabazitaxel treatment during cycles 1–2, suggesting G-CSF for neutropenia management may be critical during the early treatment cycles. Multivariate analysis of our findings showed signs of association between individual factors and early discontinuation of cabazitaxel treatment. Patient characteristics at baseline, including poor ECOG PS (≥2), high PSA levels and presence of liver lesions, indicated that patients were at a late stage of disease or had been intolerant to previous treatment. Patients with these characteristics were associated with shorter duration of cabazitaxel treatment. Our findings were consistent with earlier reports, which suggested that initiation of cabazitaxel during the late stage of disease was related to early treatment discontinuation, possibly attributed to a higher disease burden at baseline (9).

Findings from our study supported a machine learning analysis of cabazitaxel therapy in mCRPC, which suggested association of better OS with neutropenia development and higher number of cabazitaxel cycles (12). Other patient factors were dependent on the set threshold for correlation—at a higher threshold (0.05), liver metastases, ECOG PS, TTF and neutropenia were associated with OS. Apart from neutropenia, the same was true at a lower threshold (0.01) (12).

Similar to the post hoc analysis study that evaluated treatment duration of cabazitaxel among patients with mCRPC, the main reasons for discontinuation were disease progression, followed by AEs (9). Proactive management of AEs may allow patients to receive cabazitaxel treatment for longer durations and thereby achieve better outcomes (9). In our study, a greater proportion of patients who discontinued cabazitaxel treatment later (≥3 cycles) had received dose reduction upon treatment initiation. As previously reported in the PROSELICA and CABASTY studies (13, 14), this occurrence may be attributed to improvements in safety outcomes from the reduced dose, allowing longer treatment duration. However, it must be noted that patients who discontinued cabazitaxel treatment earlier (1–2 cycles) might not have received the opportunity for a dose reduction of cabazitaxel.

Although the strength of our investigation is the relatively large number of patients (>660), with a small number of patients lost to follow-up, several limitations warrant attention due to the observational and retrospective nature of our study. As this post hoc analysis was conducted on an observational PMS study, treatment regimen and patient selection were limited to the investigators' discretion, which may prompt observer bias during the data collection process, potentially contributing to erroneous values.

During the PMS, patients were not tested for genomic biomarkers, including the *BRCA* mutations. Aberrations in the *RB1* and *PTEN* genes may predict the efficacy and durability outcomes of taxane-based chemotherapy in patients with mCRPC, and subsequently guide treatment decision-making in the clinical setting (15). Future research should consider genomic biomarkers and disease stages when deciding on timing of taxane-based chemotherapy initiation and tolerability to treatment (15).

## Conclusion

Real-world data on the factors associated with early discontinuation of cabazitaxel suggest physicians may consider particular patient characteristics at baseline before initiating cabazitaxel treatment in clinical practise.

## Data Availability

This post-marketing surveillance was conducted under the Japanese Ministerial Ordinance on Good Post-marketing Study Practice for Drugs (GPSP), and due to the characteristics of the surveillance in the regulation, the scope of permission for data sharing is limited to the content described in the paper.
